# Using Health Surveillance Systems Data to Assess the Impact of AIDS and Antiretroviral Treatment on Adult Morbidity and Mortality in Botswana

**DOI:** 10.1371/journal.pone.0100431

**Published:** 2014-07-08

**Authors:** Rand Stoneburner, Eline Korenromp, Mark Lazenby, Jean-Michel Tassie, Judith Letebele, Diemo Motlapele, Reuben Granich, Ties Boerma, Daniel Low-Beer

**Affiliations:** 1 UNAIDS, Geneva, Switzerland; 2 The Global Fund to Fight AIDS, Tuberculosis and Malaria, Geneva, Switzerland; 3 Department of Public Health, Erasmus MC, University Medical Center Rotterdam, The Netherlands; 4 Yale University School of Nursing, New Haven, Connecticut, United States of America; 5 World Health Organization, Geneva, Switzerland; 6 Republic of Botswana Ministry of Health, Gaborone, Botswana; 7 Global Health Program, The Graduate Institute of International and Development Studies, Geneva, Switzerland; UCL Institute of Child Health, University College London, United Kingdom

## Abstract

**Introduction:**

Botswana's AIDS response included free antiretroviral treatment (ART) since 2002, achieving 80% coverage of persons with CD4<350 cells/µl by 2009–10. We explored impact on mortality and HIV prevalence, analyzing surveillance and civil registration data.

**Methods:**

Hospital natural cause admissions and deaths from the Health Statistics Unit (HSU) over 1990–2009, all-cause deaths from Midnight Bed Census (MNC) over 1990–2011, institutional and non-institutional deaths recorded in the Registry of Birth and Deaths (RBD) over 2003–2010, and antenatal sentinel surveillance (ANC) over 1992–2011 were compared to numbers of persons receiving ART. Mortality was adjusted for differential coverage and completeness of institutional and non-institutional deaths, and compared to WHO and UNAIDS Spectrum projections.

**Results:**

HSU deaths per 1000 admissions declined 49% in adults 15–64 years over 2003–2009. RBD mortality declined 44% (807 to 452/100,000 population in adults 15–64 years) over 2003–2010, similarly in males and females. Generally, death rates were higher in males; declines were greater and earlier in younger adults, and in females. In contrast, death rates in adults 65+, particularly females increased over 2003–2006. MNC all-age post-neonatal mortality declined 46% and 63% in primary and secondary level hospitals, over 2003–2011. We estimated RBD captured 80% of adult deaths over 2006–2011. Comparing empirical, completeness-adjusted deaths to Spectrum estimates, declines over 2003–2009 were similar overall (47% vs. 54%); however, Spectrum projected larger and earlier declines particularly in women. Following stabilization and modest decreases over 1998–2002, HIV prevalence in pregnant women 15–24 and 25–29-years declined by >50% and >30% through 2011, while continuing to increase in older women.

**Conclusions:**

Adult mortality in Botswana fell markedly as ART coverage increased. HIV prevalence declines may reflect ART-associated reductions in sexual transmission. Triangulation of surveillance system data offers a reasonable approach to evaluate impact of HIV/AIDS interventions, complementing cohort approaches that monitor individual-level health outcomes.

## Introduction

The dramatic increase in coverage of antiretroviral treatment (ART) among people living with advanced HIV infection in sub-Saharan Africa has been one of the remarkable public health achievements in the last decade [1]. By 2012, 9.7 million people in sub-Saharan Africa were estimated to be on ART, up from less than 50,000 in 2003 [2]. Local epidemiological and clinical studies in Africa have shown marked effects of ART on survival of people living with HIV, and a variety of studies ranging from community death and burial registers to demographic surveys, have demonstrated community-level impact of ART in reducing mortality [3]–[10]. Scientific evidence from observational, observational, ecological and randomized control trials suggests that ART also reduces the risk of HIV transmission during sex and a recent study in South Africa demonstrated that increases in community ART coverage levels are strongly associated with decreased individual risk [9]. Evidence of sustained countrywide population impact of ART is limited to HIV model projections [11], [12]. This is partly because most countries in sub-Saharan Africa do not have reliable birth and death registration systems with causes of death recording or hospital mortality surveillance systems [13]. South Africa is an exception, where adult mortality trends showed increases over time coinciding with HIV epidemic spread, and recent mortality declines that may be related to antiretroviral treatment [14].

Botswana has one of the most severe AIDS epidemics in the world. HIV prevalence rates among women attending antenatal clinics reached a peak of over 40% in 2003 [15]. A national household survey with HIV testing in 2004 reported adult HIV prevalence rates of 19.7% in males and 27.9% in females [16]. Modeling scenarios suggest over 190,000 cumulative AIDS deaths from 1996–2012, making AIDS the leading cause of death in adults [17].

In late 2002, the Ministry of Health initiated the ART program, which aimed to make ART freely available to eligible individuals. According to the national ART registry, the number of people currently on ART increased from 10,723 in 2003 to 50,044 in 2005, and 191,940 in 2012 [18]. An estimated 66% and 96% of individuals with CD4 cells below 200/µL were on ART by 2005 and 2009–2010, respectively (and 42% and 80% of those with CD4 cells below 350/µL) [19]. At such levels of population coverage of ART, the impact on adult mortality and morbidity would be expected to be measurable, particularly given the rapid increases in mortality and HIV/AIDS-related illness in these groups during the 1990s.

This study aimed to document the population impact of HIV/AIDS and ART on adult mortality over the course of the epidemic by analyzing two independent hospital-based morbidity and mortality surveillance systems, the civil death registry and antenatal HIV sentinel surveillance.

## Materials and Methods

Botswana has three parallel public health surveillance systems, and an ART treatment registry, that together provide data to explore temporal changes in HIV-related morbidity and mortality at the population level. We analyzed two surveillance systems that record hospital deaths and admissions through different, independent processes; a nascent civil registration system that independently records deaths in institutional settings (*i.e*. in health facilities) as well as deaths from non-institutional settings (*i.e*. outside health facilities), over 2003–2010; the Botswana antiretroviral treatment registry over 2003–2010, and HIV prevalence data from antenatal HIV surveillance over 1992–2011. Key variables, summary data and subcategories of each variable that were used for analysis, are described in Table 1.

**Table 1:** Variables used for the analysis by information source.

Information Source	Original Data Format	Years Covered	Variables Obtained and Analyzed	Dis-aggregations Analyzed
**Register of Births and Deaths (RBD)**	Unpublished PDF printouts from death certificate database	2003–2010	Deaths all ages: N = 98,614; Deaths ≥15 years: N = 85,455.	Age, sex, setting (institutional and non-institutional), year and cause of death (natural and non-natural).
**Health Statistics**	*Health Statistics Reports*	1990–2009	Deaths all ages: N = 165,975; Admissions all ages: N = 1,996,355; Natural-cause deaths, ≥15 years: N = 106,189; Natural-cause admissions, ≥15 years: N = 1,345,302.	Age, sex, setting, year of admission and death; cause of death and admission (ICD-9 or ICD-10 codes).
**Hospital midnight census**	*Health Statistics Report* for years 1990–2009; unpublished digital reports for years 2010–2011	1990–2011	Admissions and deaths ≥age 28 days from all causes. Admissions: N = 2,804,291; Deaths: N = 147,312.	Year of admission; year of death and facility type.
**ART Treatment Registry** *(MASA)*	Excel data base provided by *MASA* program	2002–2010	Patients ever and currently on ART. All ages: N = 165,654 and N = 123,474, respectively.	Age groups <15 versus ≥15 years. Cumulative by year.
**Antenatal HIV Sentinel surveillance**	Published technical report	1992–2003 (all years), 2005–2007 (3 years), 2009 and 2011	HIV prevalence among pregnant women attending antenatal care for the first time.	Age groups: 15–19; 20–24;25–29;30–34;35–39;40–49 years; calendar year and site.

### Ethics

RBD data were accessed with an Institutional Review Board permit issued to an author (ML) from the Botswana Ministry of Health's Health Research Unit. HSU and MNC data were in the form of annual Health Statistics Reports for the period 1990–2011 and were provided by the Botswana Ministry of Health; they are in the public domain. Health Statistics Reports for the period 1990–2007 used in this investigation are archived in the WHO library in Geneva, Switzerland. The antiretroviral registry data was provided by the *MASA* program, Ministry of Health Botswana, for which no permit was required.

### Data Sources

#### Health Statistics Unit morbidity and mortality reports

Morbidity and mortality recorded in institutions are reported monthly by district health teams to the Ministry of Health's Health Statistics Unit (HSU), using reporting form MoH 017 since 2003 (and a similar form in earlier years) in most cases, and in fewer cases directly from hospitals to HSU using an electronic system known as the Integrated Patient Management System. Individual records of discharges (alive or dead) contain demographic, diagnostic and discharge information, and personal identification. Non-institutional deaths are investigated by district welfare officers, reported to village chiefs, and then reported to the HSU using reporting form MoH 3002 (from 2003, and a similar form in earlier years). HSU reports the data to the Central Statistical Office. The HSU codes each discharge or death according to the WHO International Classification of Diseases (ICD, 9th or 10^th^ revision). All information from Morbidity, Mortality and Obstetric In-patient forms, including cause of death, is entered into a computerized database, which has been maintained by the HSU and the CSO since the early 1990s. The accuracy of HSU data (inpatient admission and deaths) is checked annually at the facility level for missing variables such as age, sex and diagnosis and timely reporting.

From these we analyzed nationally aggregated data on hospital admissions and discharges due to death by age, sex, and cause of death according to ICD-9 for years 1990–2003 and ICD-10 for years 2004–2009. The Statistical Report for 1993 was misplaced; therefore missing data for 1993 was imputed using simple interpolation between 1992 and 1994 data points. Age was not specified in 2% of admissions and 3% of deaths and these were imputed by redistribution of cases based on proportionate age distribution. We focused the analysis on 160,769 deaths reported in adults aged ≥15 years, of which 18,583 (12%) occurred in non-institutional settings. In order to better capture the effect of HIV-related mortality in institutional settings, we further restricted our analysis by removing data attributed to injuries (N = 4,471 deaths and N = 221,807 admissions, 63% of which were male); and deaths separately categorized as external being largely from injuries, poisoning or other adverse events with additional information regarding environmental circumstances of death (N = 3,307, 67% of which were male). These well-defined causes are unlikely to include any mis-classified HIV-related deaths, whereas the remaining natural causes of deaths, notably Pneumonia, Stroke, Undetermined cause, and Other causes, are known from earlier investigations in Botswana to often represent misattribution of HIV-related deaths – since HIV-deaths are rarely coded as HIV/AIDS.

#### Hospital midnight census

Independent of the HSU morbidity and mortality surveillance system, hospital admissions and deaths among persons aged >28 days are monitored through a daily hospital midnight bed census (MNC). These data include hospital admissions, discharge status (including deaths), and numbers of occupied beds, but without patient age, sex or causes of death. Completeness of MNC reporting is monitored with active follow-up on a monthly basis.

From annual reports published by the Central Statistical Office and unpublished reports from the Ministry of Health, we extracted numbers of admissions and discharges (live or dead) by facility for years 1990–2011. Data were summarized by year and presented from hospitals at three service levels: (1) 17 primary hospitals with a total bed capacity of 752 and 30,262 annual admissions in 2006; (2) 18 general or secondary level hospitals with a total bed capacity of 2,729 and 127, 920 annual admissions in 2006, which included (3) Botswana's two main referral hospitals, Princess Marina in Gaborone, the capital city of Botswana, and Nyangabwe in Francistown.

#### Registry of Births and Deaths

The Registry of Births and Deaths (RBD), located within the Department of Civil and National Registration in the Ministry of Labour and Home Affairs, also independently tracks and aggregates information on deaths from institutional settings, and complements these with a (near-equally large number of) death reports from non-institutional settings. Reporting of deaths is legally mandated and to be provided by medical practitioners or other persons who most recently attended the deceased. Information on decedents in health institutions includes demographic data, enumeration of symptoms at the time of death, duration of illness and hospitalization, and causes of death as certified by the medical officer of the institution in which the death occurred. If a death occurred in a non-institutional setting, only demographic data is collected and the death is then certified by the village or town chief. The completed forms (CRD-2 for institutional deaths and CRD-1 for non-institutional deaths) are sent to the RBD in Gaborone, where the information is processed into a death certificate and entered into a computerized database. A cross-sectional analysis of this registry has been published elsewhere [20].

We examined RBD-recorded mortality over the period 2003–2010 (N = 98,614, all ages combined) by age, year, setting (institutional vs. non-institutional), and district to assess reporting consistency. Data were excluded for deaths with missing age (N = 41) and from two of 41 districts (N = 4002), in which visual observations of trends indicated inconsistent reporting over the time period from 2003–2009. Non-institutional settings accounted for 40% of RBD deaths in adults and 25% of those in children from 2003 to 2010.

#### ART treatment registry and HIV antenatal sentinel surveillance

The Botswana Ministry of Health ART program (*MASA*, a Setswana word that means “new dawn”) maintains a treatment registry that provides information on persons who have ever and who are currently receiving ART by district of residence, sex, age group (<15 or ≥15 years of age), CD4 cells/µL on initiation of ART, and vital status. The program has an active follow-up system to assess patient adherence and vital status [18]. From the treatment registry, we analyzed the number of persons alive on ART by sex ages ≥15 years for the period 2003–2010.

The Ministry of Health, Botswana Ministry of Health has conducted HIV sentinel surveillance for monitoring the HIV epidemic among 15–49 year old pregnant women since 1992 according to international guidelines. Surveys have occurred on an annual basis except for the years 2004, 2008, and 2010. A detailed report summarizing the findings in 2011 and retrospective data to 1992 has been reported by the Department of HIV and AIDS Prevention Botswana [15].

#### Mortality projections by Spectrum estimation model and WHO lifetables

The Spectrum HIV policy model was developed and used by UNAIDS to estimate and project HIV-related mortality burden and treatment needs in countries with generalized HIV epidemics [12]. WHO with international health partners constructs life tables summarizing national populations' all-cause mortality patterns by age and sex, based on data reported through national civil registration systems, censuses and household surveys adjusted in a standardized for data completeness, coverage and quality [21]. We used Spectrum projections over the period 2003–2010, conducted and agreed between Botswana's NAC and UNAIDS in August 2011, and WHO life table estimates for Botswana's national population as of 2009, for comparing all-cause adult mortality against the empirical data sources.

### Analytic Plan

Because of the often poor recording of the cause of death on death certificates (with a stable 50% of causes on death certificates ill-defined) and notably underreporting of HIV deaths to allay perceived stigma, we chose to rely on the analysis of trends in mortality or morbidity from natural causes, where such disaggregation was possible, in the age groups most affected by HIV rather than those coded as HIV-related [22], [23]. From HSU data, we analyzed annual hospital admission rates per 1000 population and hospital death rates per 1000 admissions (calculated as live discharges plus deaths), and hospital deaths per 100,000 population, each by gender and age group (among adults 15 years and above) over 1990–2009, excluding admissions and deaths from non-natural causes. From MNC data, case fatality ratios (for all ages >28 days combined) were calculated using all-cause deaths as the numerator and all-cause live discharges plus deaths as the denominator. The RBD data were analyzed by age, sex, and setting (within medical institution versus outside of medical institution) for all causes and causes excluding injuries or external causes for the period 2003–2010. Corresponding hospitalizations and deaths rates were also calculated using UN Botswana population estimates [24]. We then compared temporal trends in mortality from the three mortality surveillance systems, and in HIV prevalence by age in pregnant women attending antenatal clinics with trends in persons receiving antiretroviral drugs, to determine temporal associations between reductions in deaths or HIV prevalence and increasing antiretroviral coverage.

Estimates of the number of deaths in Botswana during the course of the AIDS epidemic have varied widely [25]. According to the Botswana Central Statistics Office's Government Statistician, the accuracy of the HSU institutional death reporting when compared to population census data was estimated to be over 95% in 2001 [26]; however, WHO life table estimates that RBD data both institutional and non-institutional captured only 67% of deaths in 2003, improving to around 80% capture over 2006–2010 [21], [25]. The difference is believed to relate to underreporting of deaths in non-institutional settings [25]. While institutional reporting appears to have improved over time, the reported number of non-institutional deaths has remained constant. The proportion of HSU non-institutional deaths thus fell from 15.1% in 2005 to 4.3% in 2005 in ages 15–64 and 4.7% to 1.9% in ages 65+, remaining at the lower levels through 2009. During the same time period, however, 40% of RBD deaths in adults were reported as non-institutional and that proportion varied little over time.

We therefore estimated, and adjusted for, completeness of death registration in adults from 2003–2010 using the following approach. We combined institutional adult deaths (ages 15 years and older) from Health Statistics (HSU) and non-institutional adult deaths (ages 15 years and older) from the RBD, assuming that HSU best captures institutional deaths but largely fails to capture non-institutional deaths. We then examined the observed patterns of mortality by year from 2003–2010 in ages 65+ by sex as indicative of possible changes in reporting accuracy, since at these ages the contribution of HIV mortality is reported to be small [27]. We compared both unadjusted and completeness-adjusted empirical observed mortality trends, with trends in WHO mortality estimates and in projections of the Spectrum model.

## Results

The HSU analyses yielded a total of 106,189 hospital deaths in adults ≥15 years of age, of which there were 58,941 deaths among 497,564 admissions in males, and 47,248 deaths among 847,756 admissions in females over 1990–2009, The daily MNC enumerated 147,312 deaths among 2,804,291 hospital admissions of all ages excluding neonates over 1990–2011, and the RBD enumerated 98,614 deaths (of which N = 85,455, or 87%, were in ≥15 years) over 2003–2010.

### Health Statistics Unit: all-cause natural hospital admissions and deaths, 1990–2009

**Figure 1:**
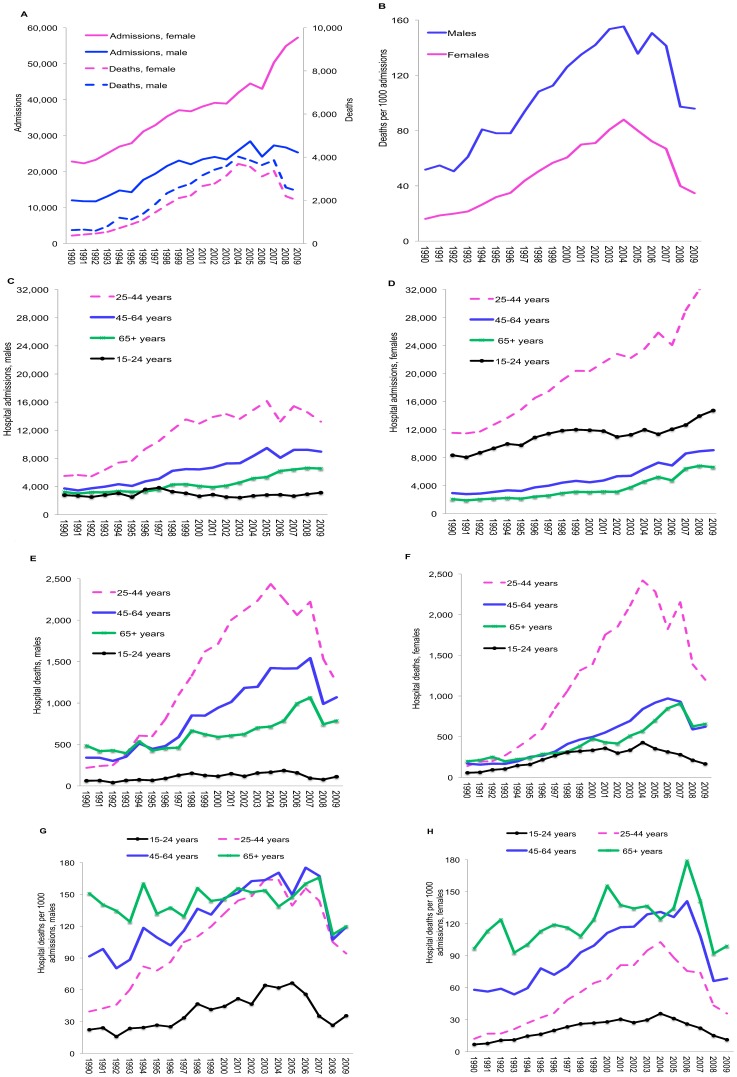
Trends in HSU-recorded hospital admissions and deaths in adults 15–64 years, 1990–2009. (A) Hospital admissions and deaths by sex; (B) Hospital deaths/1000 admissions (C) Age-specific hospital admissions, males; (D) Age-specific hospital admissions, females; (E & F) Age-specific hospital deaths, males and females; (G & H) Age- specific hospital case fatality rates, males and females.

From 1992 to 2004, HSU-recorded hospital admissions and deaths in ages 15–64 increased six- and three-fold, respectively, for males and seven- and four-fold in females (Figure 1A). From 2004 to 2007 deaths stabilized, and then declined sharply starting in 2008. Admission trends generally mirror trends in deaths through 2006, after which male admissions stabilized while female admissions continued to increase. From 2003 to 2009, hospital death rates declined by 49% (103 to 52 deaths/1000 admissions) in adults 15–64; by 55% in females (75 to 34 deaths/1000 admissions) and by 38% in males (153 to 96 deaths/1000 admissions (Figure 1B).

Among adults, the increase in hospital admissions and deaths beginning in 1992 was most marked in adults 25–44 years, the group with highest HIV prevalence (Figure 1 C–D). Admissions stabilized in 2000 in males ages 25–44 years and later in older males except in ages 65+ years, where they continued to increase. In contrast, female admissions continued to increase through 2009. This phenomenon is particularly evident in ages 25–44 years, from 2006 onwards, and relates to an increase in admissions attributed to obstetrical complications. Deaths and hospital death rates (deaths/1000 admissions) peaked and began to decline over 2004–2007; however, the magnitude and timing of declines varied and was generally greater in females and occurring later in older age groups (Figure 1 F–H). Percentage declines in death rates from 2004–2009 by age group and sex were as follows: ages 15–24, 43% in males and 68% in females; ages 25–44, 42% in males and 63% in females; ages 45–64, 30% in males and 45% in females; and ages 65+, 14% in males and 18% in females. Death rates in males were higher than in females across time in all age groups, except in 15–24 year-olds. Similar patterns are evident when hospital deaths were expressed using population sizes as the denominator, instead of admissions.

### Midnight Census: Hospital admissions and deaths, 1990–2011

**Figure 2:**
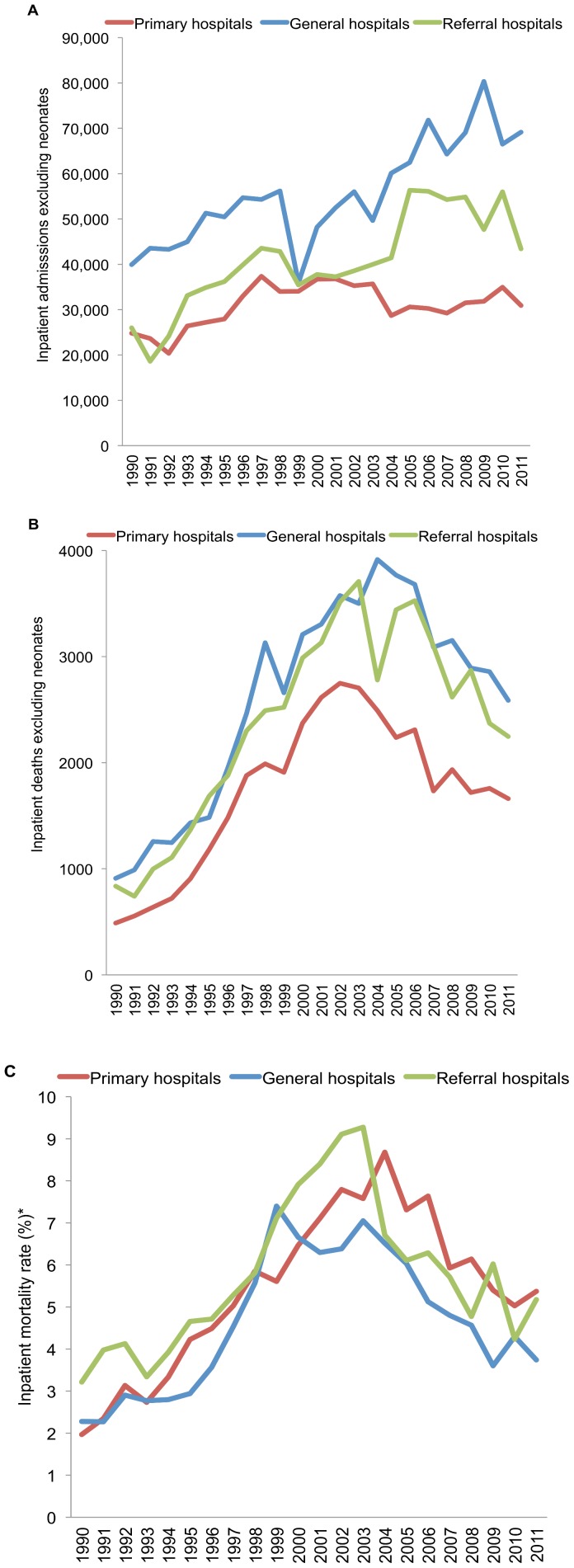
Mid-Night Census data from primary and general service category hospitals and referral hospitals (Princess Marina in Gaborone, and Nyangabwe in Francistown), 1990–2011. (A) All cause inpatient admissions excluding neonates; (B) Inpatient deaths; (C) Inpatient mortality ratio (deaths per 100 admissions).

MNC trends show similar patterns, with increasing all-cause post-neonatal hospital admissions and deaths from the early 1990s. The subsequent stabilization in admissions and decline in deaths after 2004 is most evident in primary hospitals and referral hospitals (Figure 2 A–B). In-patient mortality rates increased linearly from 1990 to a peak in 2003–4; after which they declined by over half, between 2003 and 2010, falling by 63% and 46% in general and primary hospitals, respectively (Figure 2 C).

### Registry of Births and Deaths: Institutional and non-Institutional deaths, 2003–2010

**Figure 3:**
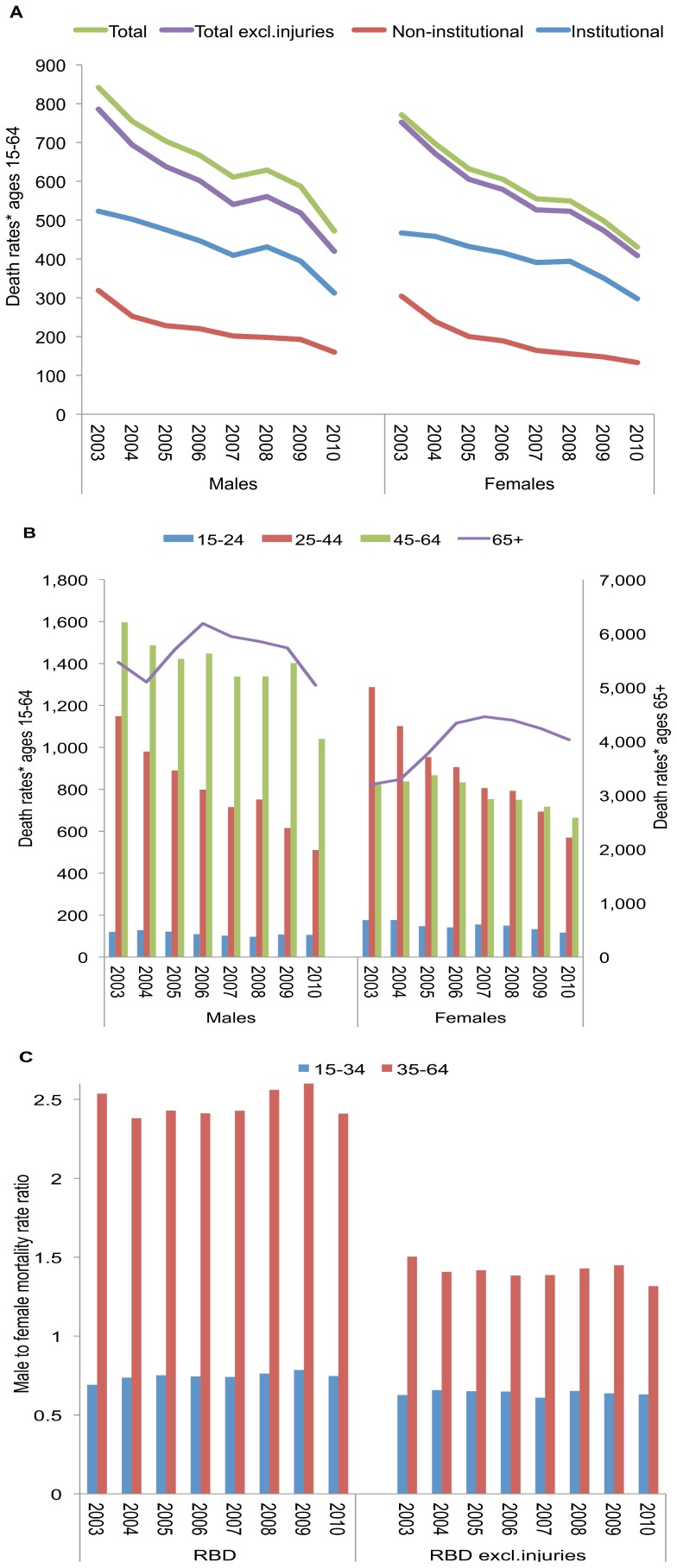
Death rates per 100,000 population* in adults, by sex and setting and male to female rate ratios, Registry of Births and Deaths (RBD), 2003–2010. (A) RBD total, RBD excluding injuries and external causes, institutional, and non-institutional setting, by sex, for ages 15–64; (B) RBD death rates by age and sex; (C) Ratio of male death rates to female death rates ages 15–34 and 35–64.

A total of 85,455 adult (age 15+years) deaths were reported by the RBD over 2003–2010: 52% and 48% were in males and females, respectively; and 60% and 40% occurred in institutional and non-institutional settings. The distribution of deaths by sex and setting remained relatively constant over the period. All-cause mortality rates in 15–64-year-olds, as well deaths with known injury causes excluded, declined by 44% and 46%, respectively over 2003–2010, and similarly in males and females (Figure 3A). Institutional and non-institutional deaths declined 38% and 52%, respectively. Declines were earlier and greater in ages 25–44 compared to ages 45–64, and were similar by setting and exclusion of injury related causes (Figure 3B; Figure S1). In contrast, death rates increased over 2003–2010 for adults 65+ years, by 35% for females and by 13% for males and particularly over 2003–7 (Figure 3B), without difference between institutional and non-institutional deaths (Figure S1). The average male-to-female ratio in death rates for RBD data overall and with injury causes excluded were 111 males/100 females and 105 males/100 females, respectively, for ages 15–64; however when stratified by age (at *a priori* chosen cut-off of 35 years), ratios in ages 35–64 years were over twice those of younger adults (Figure 3C).

### Temporal Association of trends in mortality, antiretroviral use and HIV prevalence from antenatal HIV surveillance

**Figure 4:**
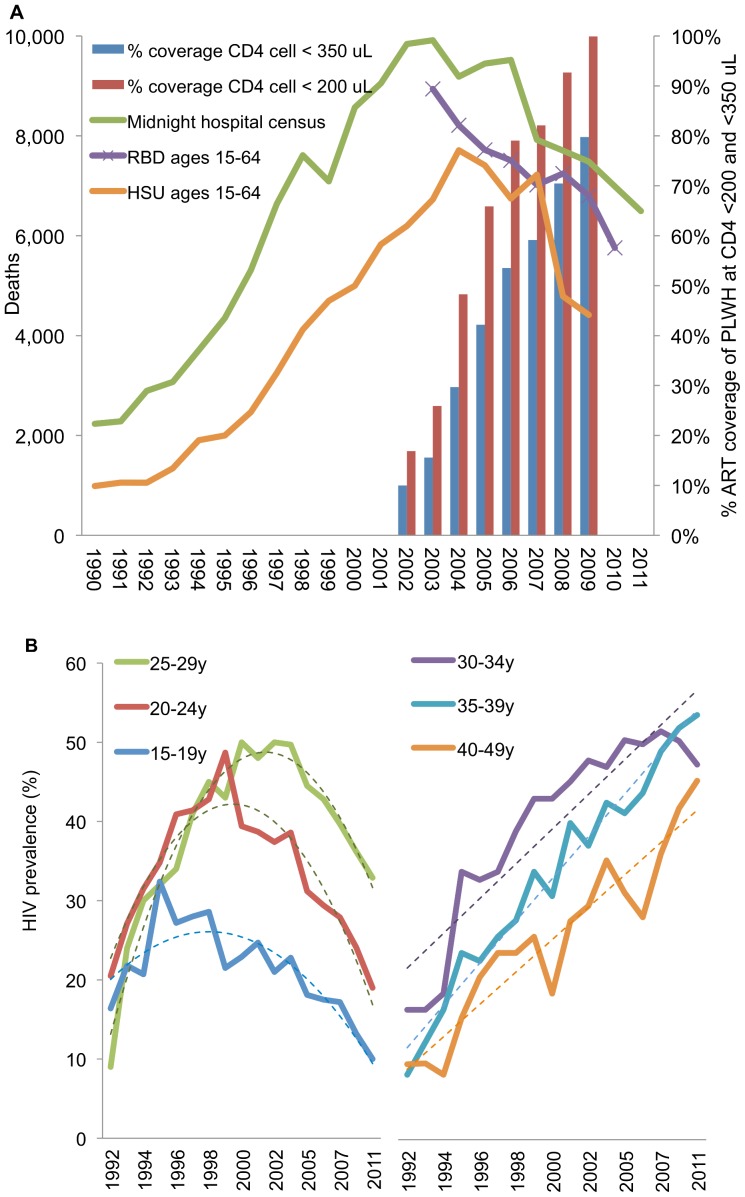
Trends in deaths, ART coverage rates and HIV prevalence among pregnant women. (A) Deaths reported from Health Statistics (HSU; institutional natural-cause deaths only), Hospital Midnight Census (MNC), and Registry of Births and Deaths (RBD; institutional and non-institutional, including non-natural causes) and estimated ART treatment coverage rates of persons ≥15 years with CD4 cells <200/µL and <350/µL, 1990–2011; (B) HIV prevalence by age group among pregnant women from the antenatal sentinel surveillance system, 1992–2011. Dashed lines represent best fitting quadratic (left) and linear (right) curves.

Trends in reported deaths from the three public surveillance systems and numbers of persons receiving ART over 1990–2011 are summarized in Figure 4A. Mortality crested in 2003–2004 and declined considerably through 2009–2011: all-cause MNC deaths in ages >28 days declined 34% from 2003 (N = 9,923) to 2011(N = 6,537); all-cause RBD deaths in ages 15–64 declined 36% (from N = 8,940 in 2003 to 5,757 in 2010); all natural cause HSU institutional deaths declined 43% (from 7,712 in 2004 to 4,414 in 2009).

In comparison, trends in HIV prevalence from antenatal sentinel surveillance sites over 1992–2011 (Figure 4B) among 15–29 year olds illustrate a gradual epidemic reversal, from epidemic spread through stabilization into decline, which persisted and accelerated after start of ART scale-up. In the age groups with highest incidence of new infections, prevalence declines started in the late 1990s for 15–24 year-old women and around 2000–2003 for women 25–29 years − and these declines persisted after 2003 (fitted, for all 3 groups, by quadratic models, with calendar year having both a significant linear positive as well as a quadratic negative effect, R2>0.70, with p<0.01 for the quadratic term in all 3 groups).

In contrast, for women above 30 years – in whom prevalence trends mainly reflect survival after earlier HIV infection, rather than new infections – prevalence continued to rise throughout the 2000s (fitted by linear time trends throughout 1992–2011, at R2>0.77, p<0.002 for all 3 groups – without any strong quadratic effects).

### Completeness of mortality capture and comparisons to modeled mortality trends

Comparison to WHO life table estimates suggests that completeness of death reporting in the RBD for ages ≥65 years increased somewhat over the time period evaluated, at 66% in 2003–04, 74% in 2005, and between 79% and 82% over 2006–2010. Assuming that completeness in reporting had improved similarly over time for younger adults, RBD records would thus underestimate the mortality declines over 2004–2010. However, a substantial difference between rate increases in females ages ≥65 years compared to those of males, raised concerns that some of the observed death rates in ages ≥65 years, particularly evident from 2003–2006, may be confounded by limited access to care or other disease-related factors in addition to improved reporting. Given this concern we applied a conservative annual adjustment factor for underreporting over the period 2003–2006, based on the average WHO-estimated reporting completeness ages 15–64. This adjustment factor changed the assumed reporting capture in adults from an average of 60% from 2003–2005 to 87% by 2009.

**Figure 5:**
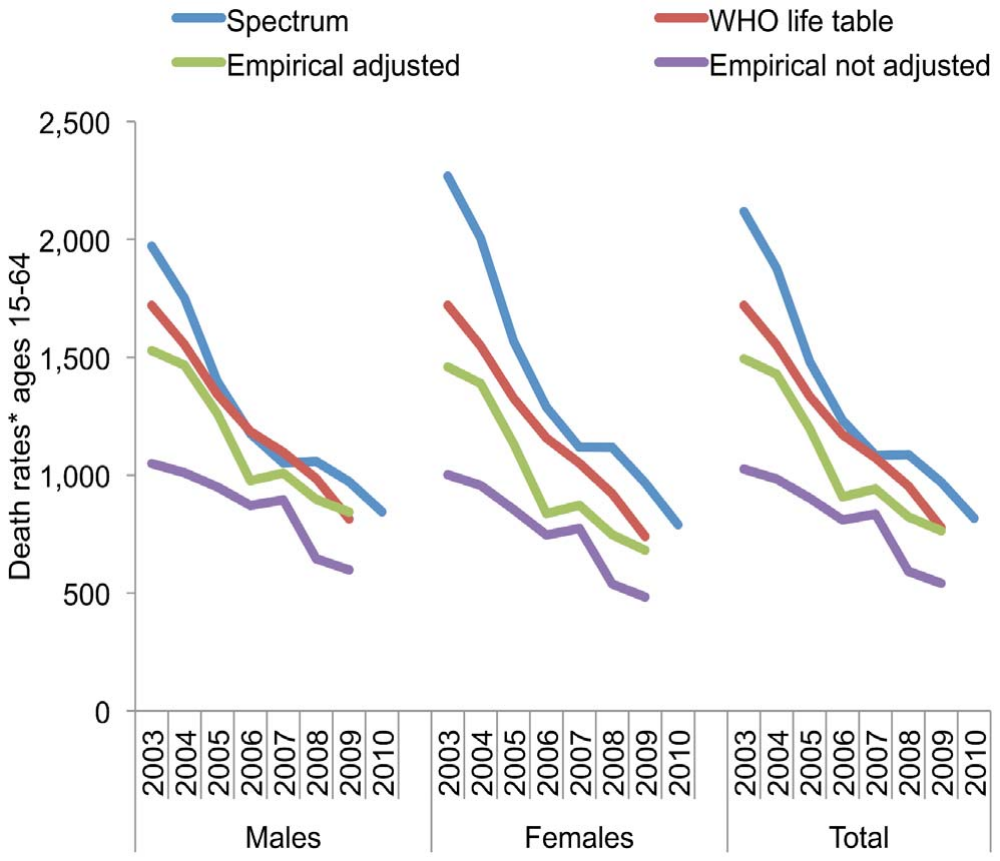
Comparison of trends in all-cause death rates per 100,000 population in ages 15–64 by sex and total, between Spectrum epidemiological projection model, WHO life-tables, and empirical surveillance data (RBD+HSU combined) with and without adjustments for estimated reporting completeness.

After adjusting trends in mortality rates for lower capture of non–institutional deaths and potential underreporting, mortality declines from 2003 to 2009 changed from an unadjusted 33% and 47% in RBD and HSU data, to 47% in the empirical adjusted estimate (HSU and RBD). This adjusted decline was still slightly less than declines estimated by the WHO life table (55%) and the Spectrum projection model (54%) over the same period (Figure 5 A). Both modeling methods estimated slightly larger declines in women than in men, but these differences reduced in the late-2000s. Among the three sources, Spectrum estimated the largest cumulative number of all-cause deaths.

The scale of death rate declines in ages 25–34 years is similar across the three sources. However, Spectrum estimated relatively higher death rates and numbers in both men and women, and declines of greater magnitude and occurring earlier, particularly in women, compared to other sources (Figure S2 A–C). The average male-to-female death ratios from 2003–2009 for the RBD data and Spectrum were 106 males/100 females and 95 males/100 females, respectively, for ages 15–64. Ratios in ages 35–64 were substantially higher (33%–48%) than RBD based estimates than those generated by Spectrum (Figure S2 D).

## Discussion

The ability to evaluate impact of nation-wide disease control programs using ecological analysis is constrained by the inevitable lack of a no-program control group. Although this analysis of trends across three surveillance systems cannot provide causality, the strength and consistency of time trends across the independent systems and the close temporal relationship of mortality declines with intervention scale-up provide strong plausibility of the impact of first the spread of HIV and then the HIV response, including ART, on adult mortality trends in Botswana. Hospital morbidity and mortality rates increased five-fold from 1990–2003 among age groups most affected by HIV infection [28]. Co-incident with the rollout of ART in 2003, hospital deaths stabilized and then declined, most markedly in young adults (15–29 years) and in females. Hospital mortality declines were corroborated by an independently collected nightly bed-census system that indicated a 55% decline in hospital case fatality rates over 2003–2011. Declines of similar magnitude were evident in civil registration data, again most marked in young adults. The association of increases then declines in mortality evident in age groups most affected by HIV and the subsequent decline coinciding with ART uptake offers is consistent with an effect of ART in prolonging survival among persons with HIV immune-suppression. The magnitude of mortality declines associated with ART treatment is similar to that earlier observed in higher-income settings [29], [30].

Earlier ongoing declines in HIV prevalence, related to prevention or natural epidemic dynamics, and increased uptake of ART have likely contributed to the mortality decline in young adults, which was more marked than declines in older age groups. The mortality pattern mirrors, with several years delay, trends in HIV prevalence, which in pregnant women aged 15–24 fell by 60% over 1998–2011, and by 34% in pregnant women aged 25–29 years from 2003, whereas HIV prevalence stabilized or continued to increase in older pregnant women [15], [16], [31]. The apparent acceleration in HIV prevalence declines in 15–29 year-olds over 2003–2011 may relate to concurrent ART scale-up, which should reduce viral load and sexual transmission efficiency [9], [32]. Although reductions in high risk sexual behaviors may have contributed to reductions in HIV incidence in this age group, evidence from population surveys suggests that that levels of risk behaviors have not changed dramatically since 2001 [15], [31]. Furthermore, the coincident drop in prevalence particularly in ages 20–29 suggests an association with common external exposure effect (i.e., ART effect on HIV incidence) compounding the ongoing natural dynamics and cohort ageing. Comparison of age-specific HIV prevalence between Botswana's fourth national HIV/AIDS population survey (BAIS 2012) and similar representative household surveys conducted in earlier years will provide important evidence to explore this hypothesis [33].

Biases related to data quality in terms of completeness and coverage often limits analysis and interpretation of surveillance, survey or civil registration data. The accuracy of cause of death from these sources was of limited quality; therefore, we focused our analyses on trends in adult mortality from all natural causes by age and sex. The observed patterns of mortality were similar to those reported for AIDS cases from other settings and generally similar to projections from epidemiological models [28], [30]. However there were notable differences: magnitudes of mortality decline in ages 15–64 were smaller and later in the empirical data compared to model projections, especially for older adults, and for ages 35–64 years the surveillance systems recorded more deaths in males than in females, whereas Spectrum projected male-to-female mortality rate ratios below 1 (Figure S2 D). The differences between observed and modeled mortality could relate to biases associated with variations in reporting completeness by age, sex and calendar year– which our adjustments to the empirical data may not fully have solved. In addition, imprecise modeling assumptions regarding the timing and peak in HIV incidence, or other HIV dynamics parameters – shared between WHO life-tables and Spectrum projections – may explain differences [34]. Spectrum projections may overestimate HIV incidence and resulting mortality rates in younger age groups, as it assumes relatively static age-specific HIV-incidence over time [28].

The lag in mortality decline observed in older age groups and the persistent high mortality in males, evident in RBD and HSU, are less likely related to data quality issues; however, they may be plausibly explained by differential access to HIV testing, counseling and ART by age and gender. A national household survey performed in 2008 indicated that 41% of men aged 15–64 had never been HIV-tested, compared to only 25% of females, while in the same year among 61,314 persons who reported accessing ART services, 32% were male and 68% were females [31]. Indeed, health-care seeking is lower in males than in females, as males in general don't access public primary health services afforded women through reproductive health services. Other contributors to the lesser mortality declines in men may include social, host and lifestyle factors that place males and older adult males at higher risk for poor health outcomes, for HIV/AIDS as well as common chronic diseases such as diabetes, hypertension, heart disease, stroke and renal failure [35]–[40].

Also the recorded increase in mortality over 2003–2006 in ages 65+, notably in females and evident in both institutional and non-institutional settings, is difficult to attribute entirely to improving reporting completeness, for the following reasons: HIV prevalence was above 10% in the 65+ age group in 2003: the proportion of HIV-attributed reported deaths (albeit largely undercounted) in these ages more than doubled between 2003 and 2010, while deaths from other causes remained stable, and HIV deaths in younger ages declined. High risk for HIV infection and increasing mortality in ages 65+ has been reported in Kenya, and poorer clinical outcomes for older people on ART have also been reported elsewhere [41]. The temporal relationship of this finding to early ART roll-out and its predominance in females may relate to treatment complications such as the ART metabolic syndrome, risk factors of which include older age and obesity, the latter being more common in Batswana females than males [42], [43].

All in all, reporting biases are unlikely the predominant explanation for the mortality shifts observed in Botswana's national surveillance systems, given the similarity of initial rises and subsequent declines across the three independent monitoring systems, and their consistency with morbidity and mortality age patterns and trends typical of AIDS [28], [44]–[46], and the close temporal association of onset of declines with the roll-out of ART.

The triangulation approach demonstrates the value of evaluating program impact from basic epidemiological and ecological analysis of multiple health surveillance data, even those considered of imperfect quality, noting that limitations frequently associated with observational data (i.e. the ecologic fallacy) may often be overstated [48]. According to WHO life-table estimates, the RBD civil registration captured no less than 80% of estimated adult deaths over 2006–2010, and possibly more given uncertainties in the demographic modeling approaches. Triangulation among several routine data sources can provide a critical basis to evaluate the impact of evolving disease trends including HIV/AIDS and to highlight emerging risks that may not be identified by periodic surveys, one-off small-scale studies or epidemiological modeling. As biomedical interventions assume an increasingly dominant role in HIV/AIDS control, refining surveillance systems that report hospital HIV-related morbidity and mortality by ART treatment status and improving their timely reporting would be an important adjunct to current ART impact evaluation systems (i.e. based on ART treatment registries, program output data and epidemiological simulation modeling). AIDS surveillance systems served as the foundation for monitoring AIDS and HIV impact in the past and could be useful again in identifying differential coverage and impact of interventions across populations and geographic areas, providing more direct empirical evidence of program impact than modeling approaches. Furthermore, examination of hospital-level surveillance data can provide insight to potential differential disease trends (HIV and non-HIV) by time, age or gender, informing emerging health risks or inequalities in care that can better guide health policy and research. Improved mortality estimates will stem from formal validation of the Botswana mortality reporting systems, including comparisons with mortality patterns with neighboring South Africa, which had similar HIV dynamics but a high capture rates of adult mortality over the past two decades [47], as well as with mortality statistics data from Botswana's 2011 population census [48].

Our findings and methodological approach may not be generalizable to other countries in sub-Saharan Africa. Botswana benefits from the highest socioeconomic status in sub-Saharan Africa, a superior health care infrastructure and an aggressive early response to provide ART to nearly everyone eligible under WHO recommendations. [18]. Few countries in sub-Saharan Africa have functional vital registration or Health Management Information Systems like Botswana's; and only South Africa has a vital registration that meets international quality standards. Nevertheless, countries are improving their systems and many countries now have some system of morbidity and mortality monitoring that may be useful for managing and evaluating health events and interventions [49].

In summary, our findings indicate that the benefits of ART in reducing adult deaths in Botswana may be of similar scale to that in more developed countries. The co-incident accelerated decline in HIV prevalence in pregnant women may reflect an ART-related reduction in HIV incidence at the population level. The magnitude of mortality reduction corroborates empirical evidence to confirm progress in achieving The United Nations 2015 Millennium Development Goal 6 of reducing AIDS deaths by 2015, and to set reasonable targets there beyond [50].

## Supporting Information

Figure S1 (A–B): Death rates per 100,000 populations* in adults ages 15–64 by sex and setting, Registry of Births and Deaths, 2003–2010; (A) Institutional setting; (B) Non-institutional setting.
(PDF)

Figure S2 (A–D): Comparison of trends in estimates of adult all cause death rates per 100,000 population generated by the Spectrum model, the WHO life-table methods, and empirical data with and without adjustments for changes in reporting, 2003–2010; (A) Death rates ages 15–34 by sex; (B) Death rates ages 35–64 by sex; (C) Proportionate distribution of annual deaths by age group and sex; (D) Ratio of male death rates to female death rates.
(PDF)
