# Supplementary figures and images for: Using Health Surveillance Systems Data to Assess the Impact of AIDS and Antiretroviral Treatment on Adult Morbidity and Mortality in Botswana
Source: PLoS One. 2014 Jul 8;9(7):e100431. doi: 10.1371/journal.pone.0100431 (PMC4086724; doi:10.1371/journal.pone.0100431)

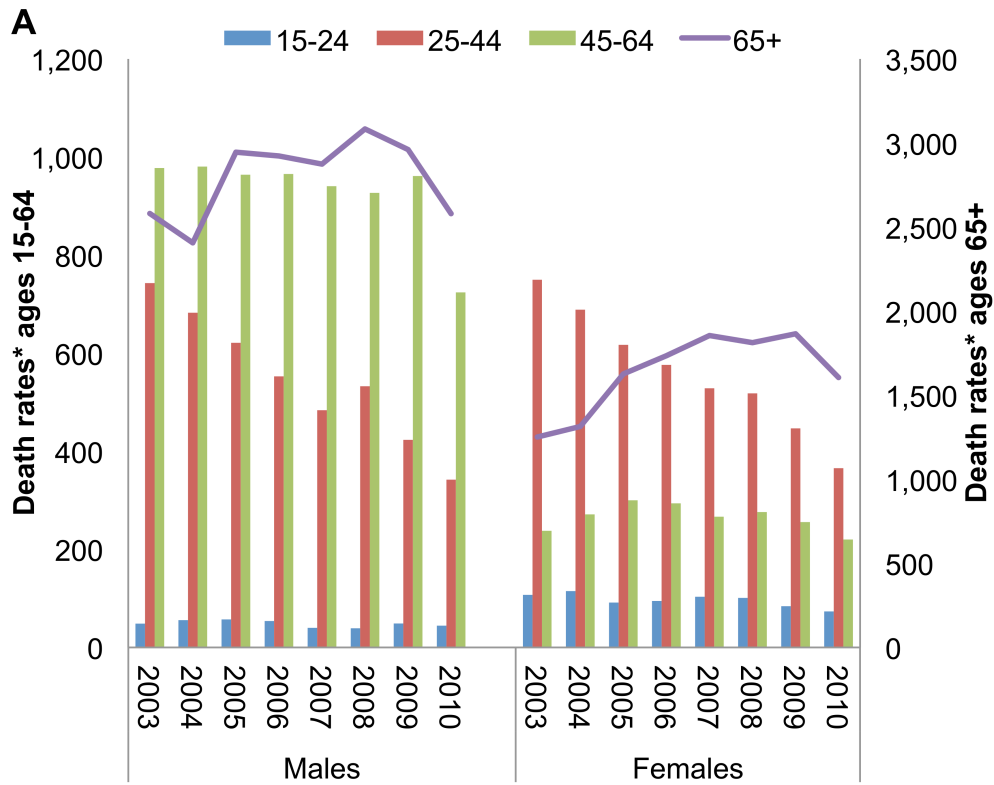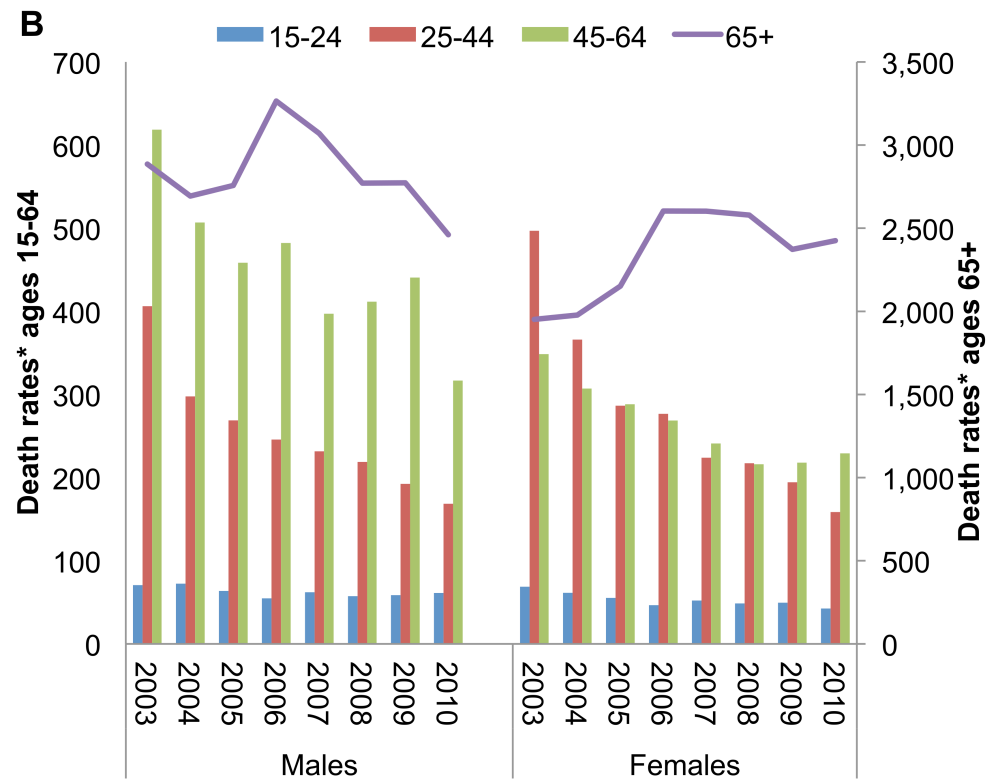

Supplement: Figure S1 — (A–B): Death rates per 100,000 populations* in adults ages 15–64 by sex and setting, Registry of Births and Deaths, 2003–2010; (A) Institutional setting; (B) Non-institutional setting. (PDF) [file pone.0100431.s001.pdf]
