# Supplementary material for: Using Health Surveillance Systems Data to Assess the Impact of AIDS and Antiretroviral Treatment on Adult Morbidity and Mortality in Botswana
Source: PLoS One. 2014 Jul 8;9(7):e100431. doi: 10.1371/journal.pone.0100431 (PMC4086724; doi:10.1371/journal.pone.0100431)
Supplement: Figure S2 — (A–D): Comparison of trends in estimates of adult all cause death rates per 100,000 population generated by the Spectrum model, the WHO life-table methods, and empirical data with and without adjustments for changes in reporting, 2003–2010; (A) Death rates ages 15–34 by sex; (B) Death rates ages 35–64 by sex; (C) Proportionate distribution of annual deaths by age group and sex; (D) Ratio of male death rates to female death rates. (PDF) [file pone.0100431.s002.pdf]

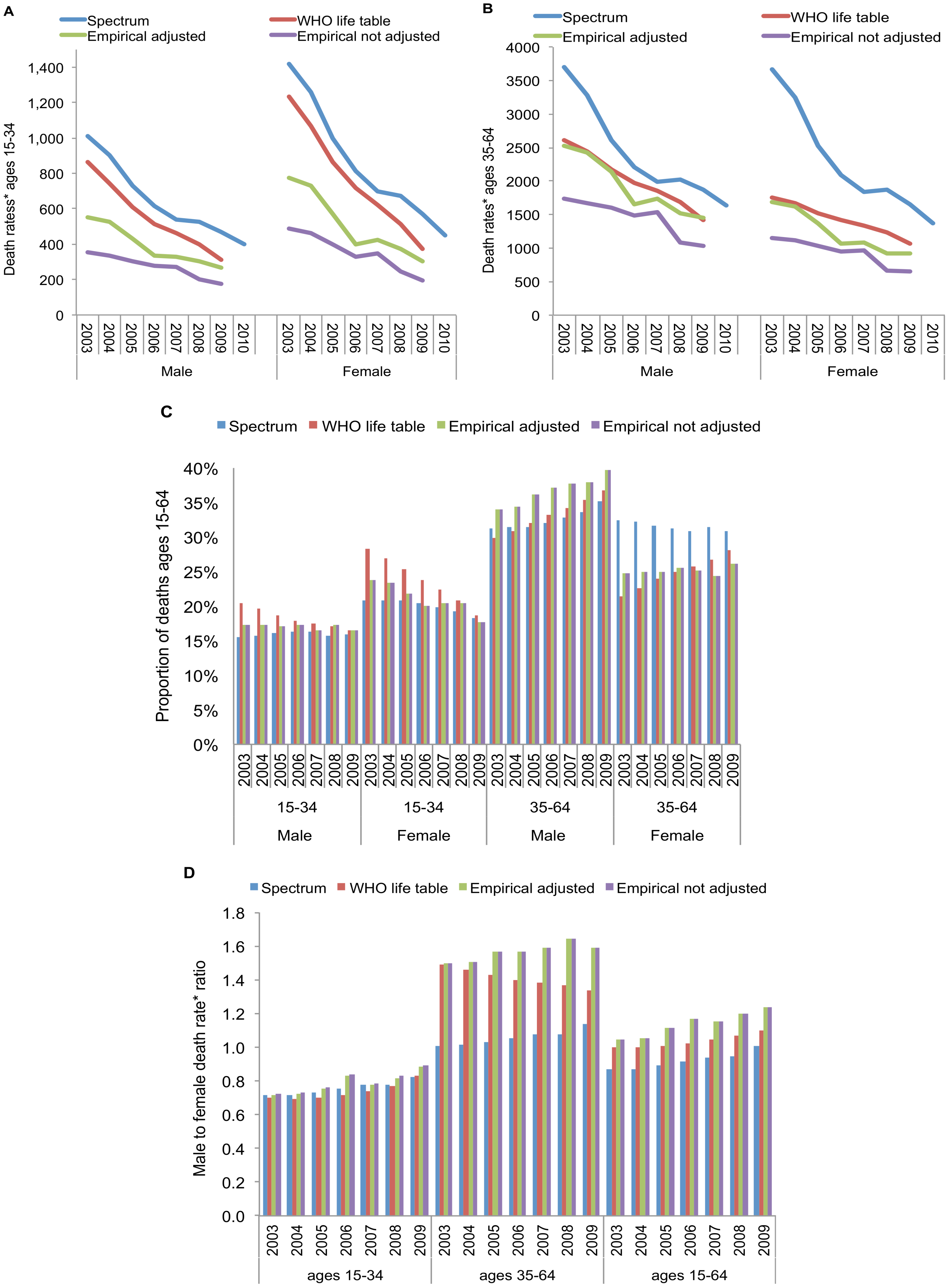

A
Spectrum
Empirical adjusted
WHO life table
Empirical not adjusted
Death ratess* ages 15-34
0
200
400
600
800
1,000
1,200
1,400
2003 2004 2005 2006 2007 2008 2009 2010
Male
2003 2004 2005 2006 2007 2008 2009 2010
Female
B
Spectrum
Empirical adjusted
WHO life table
Empirical not adjusted
Death rates* ages 35-64
0
500
1000
1500
2000
2500
3000
3500
4000
2003 2004 2005 2006 2007 2008 2009 2010
Male
2003 2004 2005 2006 2007 2008 2009 2010
Female
C
Spectrum
WHO life table
Empirical adjusted
Empirical not adjusted
Proportion of deaths ages 15-64
0%
5%
10%
15%
20%
25%
30%
35%
40%
2003 2004 2005 2006 2007 2008 2009
15-34
Male
2003 2004 2005 2006 2007 2008 2009
15-34
Female
2003 2004 2005 2006 2007 2008 2009
35-64
Male
2003 2004 2005 2006 2007 2008 2009
35-64
Female
D
Spectrum
WHO life table
Empirical adjusted
Empirical not adjusted
Male to female death rate* ratio
0.0
0.2
0.4
0.6
0.8
1.0
1.2
1.4
1.6
1.8
2003 2004 2005 2006 2007 2008 2009
ages 15-34
2003 2004 2005 2006 2007 2008 2009
ages 35-64
2003 2004 2005 2006 2007 2008 2009
ages 15-64
